# Defining a minimum nodal yield for neck dissection in mucosal head and neck squamous cell carcinoma, a systematic review

**DOI:** 10.1007/s00405-025-09250-x

**Published:** 2025-02-21

**Authors:** Sara Bassani, Paolo Tesauro, Daniele Monzani, Gabriele Molteni

**Affiliations:** 1 Otolaryngology-Head and Neck Surgery Department, University of Verona, Verona, Italy; 2 Department of Otolaryngology, Head and Neck Surgery, IRCCS Azienda Ospedaliero-Universitaria Di Bologna, Bologna, Italy; 3 Department of Medical and Surgical Sciences, Alma Mater Studiorum, University of Bologna, Bologna, Italy

**Keywords:** Lymph node yield, Neck dissection, Head and neck squamous cell carcinoma, Prognostic indicator, Standardization

## Abstract

**Background:**

Analysis of existing literature on lymph node yield (LNY) in neck dissection (ND) for head and neck squamous cell carcinomas (HNSCC) used as a prognostic factor and an indication of treatment adequacy.

**Methods:**

PubMed, EMBASE and Web of Science databases were systematically searched from January 2010 to June 2023. Inclusion criteria encompassed studies on mucosal HNSCC patients undergoing ND with data on LNY and its association with survival outcomes. The quality assessment followed the REMARK guidelines.

**Results:**

Among 29 included studies, minimum LNY tresholds associated with improved survival outcomes ranged from 10 to 36.5 nodes. The heterogeneity in subsite involvement and cN0/cN + status constituted a challenge in establishing a consensus cutoff. The review highlights the need for standardized surgical techniques and pathological assessments to ensure data comparability.

**Conclusions:**

LNY is a prognostic indicator and could reflect ND quality in HNSCC.

## Introduction

Head and neck squamous cell carcinomas (HNSCC) develop from the mucosal epithelium in the oral cavity, pharynx and larynx and are the most common malignancies that arise in the head and neck [1]. Regional lymph node (LN) metastases are associated with increased mortality in HNSCC, indeed the involvement of cervical lymph nodes is among the most important independent prognostic factors.

The 8th AJCC pathologic TNM staging system considers for nodal staging the number of positive nodes, size, laterality, and the presence of extra-capsular spread (ECS). On the one hand the current classification is effective for directing the need for additional therapy, on the other hand current staging may underestimate the cumulative effect of escalating metastatic nodal burden and it also fails to consider the quality of neck dissection.

There is mounting evidence that the absolute number of pathologically positive LNs may be more important for prognosis than other factors. Several studies have suggested that pathologically positive LNs can be used to create a simple, objective, and more accurate nodal classification system than the current AJCC system for oral cavity, larynx, hypopharynx and oropharynx [2–6]^.^ Recent studies have shown that overall mortality escalates continuously along with the increased number of positive LNs [2] and it is also associated with a decrease in the number of LNs retrieved from neck dissection in patients with head and neck cancer [7].

The total number of lymph nodes excised during a surgical resection of a tumor constitutes the lymph node yield (LNY). The LNY from regional nodal dissection in surgically treated patients has emerged in recent years as a quality metric that correlates with long-term survival and it has shown a significant prognostic capacity in different tumor models, including carcinomas of the bladder [8], breast [9], colorectal [10], gastric [11], or esophageal [12]. In colorectal cancer (CRC) a minimum number of 12 LNs examined after oncologic resection has been found to correlate with improved survival and has been included in the National Comprehensive Cancer Network evidence-based guidelines for CRC.

Regarding the neck dissection for HNSCC a directly proportional correlation between the number of lymph node metastasis and LNY in the surgical specimen has been demonstrated. Several recent studies have found an association between the number of LNs examined from a neck dissection and overall survival in both patients who are node negative and node positive with head and neck cancer [13, 14]. Although these findings a minimum LNY to define an adequate neck dissection for patients with HNSCC has still not been reached.

The aim of the article is to review the current literature regarding LNY in HNSCC in order to obtain a proposal for a minimum number of lymph nodes that should be retrieved during neck dissection in HNSCC that define the quality of a neck dissection and its correlation with survival of the patients.

## Methods

### Search strategy

We strictly adhered to the Preferred Reporting Items for Systematic Reviews and Meta-Analyses (PRISMA) [15] guidelines.

For our search strategy, we conducted a thorough systematic search of articles published between January 2010 and June 2023 in the PubMed, EMBASE, and Web of Science databases with the combined query: “(cancer OR carcinoma OR neoplasm) AND (head and neck OR oral OR oropharynx OR larynx OR hypopharynx OR nasopharynx OR mouth OR gingival OR lip OR tongue OR palate OR otorhinolaryngology OR lingual OR tonsil) AND (yield OR count OR distribution OR number OR amount) AND (lymphnode OR node OR nodes OR nodal) AND (neck dissection OR selective neck dissection OR radical neck dissection OR modified neck dissection) AND (English) NOT thyroid NOT salivary glands NOT esophagus NOT skin”.

Subsequently, the full text of relevant studies was screened for final selection. All studies identified by the initial literature search were reviewed independently by two authors (SB and PT). All titles and abstracts were assessed. When in doubt, the full text was scrutinized, and if a dispute remained, it was resolved by a senior author (GM).

We aligned our assessment with the Patient, Intervention, Comparison, Outcome, and Study Design (PICOS) guidelines, with a primary focus on patients with mucosal squamous cell carcinoma of the head and neck who underwent neck dissection as their primary treatment. The intervention of interest was the number of lymph nodes retrieved during neck dissection (LNY), which served as both the intervention and the basis for comparison. Our primary clinical outcomes of interest were overall survival (OS), disease-specific survival (DSS), and disease-free survival (DFS).

### Inclusion and exclusion criteria

The inclusion criteria are as follows:

- Articles published between January 2010 and June 2023;
- Patients affected by mucosal head and neck SCC confirmed by histopathology;
- Patients undergoing surgical treatment that includes neck dissection without any previous treatment;
- Data on LNY, defined as the total number of nodes retrieved histologically during neck dissection;
- Data on LNY and its associated oncological outcomes.

The exclusion criteria are as follows:

- Duplicate articles;
- Articles in a language other than English;
- Book chapters, case reports, poster presentations;
- Articles analyzing other than SCC head and neck histologies;
- Previous radiotherapic treatment;
- Articles on thyroid cancer, head and neck skin cancer, cervical esophagus cancer and salivary glands cancer;
- Absence of oncological outcomes.

### Data collection

Regarding data extraction, we collected information such as the first author, publication year, study location, the number of patients, patient demographics (age and gender), involved subsites, type of neck dissection with involved levels, total nodal yield, and survival outcomes related to nodal yield. We also recorded follow-up duration and TNM staging according to various editions of the AJCC staging system [16].

The survival endpoints were either OS, DSS and/or DFS. OS was defined as the time between the date of surgery and date of death or last follow-up, DSS as the time between the date of surgery and date of cancer-related death or last follow-up, and DFS as the time between the date of surgery and the date of local/ nodal/distant recurrence or last follow-up.

### Appraisal of study quality

We adhered to the reporting recommendations for tumor prognostic studies (REMARK) guidelines. REMARK involves eight different domains. Each domain is considered adequate (1) or not adequate (0) [17]:

- Well-defined inclusion and exclusion criteria
- Nature of the study (prospective or retrospective)
- Patient characteristics described
- Tumor characteristics described
- Lymph node yield measurement described
- Study endpoints or outcomes described
- Follow-up period described
- Patients unavailable for statistical analysis identified (i.e., lost to follow-up)

We gave a total score from 0 to 8 to each study, indicating the lowest and the highest quality, respectively. A total score > 4 was considered globally adequate.

### Synthesis

Given the variation in results among the studies, a quantitative meta-analysis was not feasible, precluding a comprehensive pooled analysis. We meticulously examined the included studies for practical clinical relevance, considering factors such as tumor site, lymph node status (cN0 or cN +), extent of ND, bilateral NDs, the rationale behind chosen cutoff levels, and the specific survival metrics employed in the analyses.

## Results

**Fig. 1:**
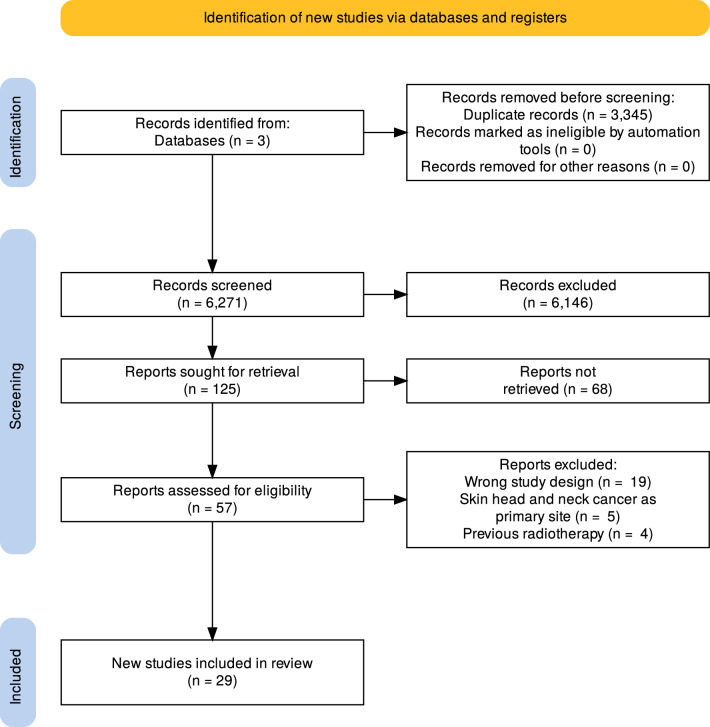
PRISMA

In the initial phase of our literature review, we meticulously examined 6271 articles, excluding duplicates. After thoroughly evaluating titles and abstracts, 125 articles were subjected to full-text screening to determine their eligibility. Ultimately, 29 articles met our inclusion criteria (Fig. 1).

The selected studies provide comprehensive insights into patients diagnosed with SCC in various anatomical regions, including the larynx (8 articles), oral cavity (21 articles), oropharynx (9 articles), hypopharynx (4 articles), and paranasal sinuses (1 article). Notably, two studies conducted by Koehler et al. [18] and Feng et al. [19] concurrently address patients with SCC located in the oral cavity and oropharynx. Additionally, the investigations by Divi et al. [14], Merz et al. [20], and Leon et al. [21] collectively encompass patients with SCC affecting the larynx, oral cavity, oropharynx, and hypopharynx. Butt et al. [22], in contrast, consider patients with SCC across the larynx, oral cavity, oropharynx, hypopharynx, and paranasal sinuses simultaneously.

All identified studies adopt a retrospective approach, with most being multicentric. Several studies utilize the National Cancer Data Base (NCDB), an extensive repository encompassing data on cancer patients across the United States [23]. Notably, the NCDB studies exhibit varying sample sizes, potentially leading to overlaps in their respective study populations. Furthermore, some investigations rely on data from the Surveillance, Epidemiology, and End Results (SEER) database [24], which is designed to represent the overall U.S. population. Given the nature of these databases, there is a plausible likelihood of overlap between the SEER and NCDB datasets.

The included studies take into consideration both cN0 and cN + patients.

### Quality assessment

Assessing the studies based on the REMARK criteria [17] revealed a quality range from 4 to 7, with a median score of 6. Out of the 29 included studies, 27 scored above 5, indicating that most of the studies could be categorized as moderate quality. The two studies that were not judged adequate were excluded.

### Identified LNY cutoff values

These studies’ identified cutoff values vary, revealing distinct associations with survival outcomes. Some studies did not identify proper cutoffs associated with better survival outcomes.

Koehler et al. [18] suggest a cutoff of 35, correlating with an 80% correct staging rate and a notable impact on survival, particularly for early-stage. Ho et al [3] propose a similar cutoff of 35, demonstrating a continuous decrease in the risk of death with each additional harvested node.

Amar et al. [25] advocate for a cutoff of 30, highlighting improved DFS with the retrieval of 30 or more nodes. Shah et al [26] propose a lower cutoff of 10, showing a correlation between enhanced OS and reduced locoregional relapses.

Tsai et al. [27] identify 23 as the cutoff, associated with higher 5-year OS. Feng et al [19] propose a cutoff of 20, indicating survival benefits from adjuvant radiotherapy in tongue cancer patients when the LNY is under the value of 20.

Lee et al. [28] find a cutoff of 19, where a higher LNY is linked to better OS and DSS. Merz et al [20] establish 15 as the cutoff, which is significantly associated with improved OS in cN0 patients.

Zenga et al. [29] pinpoint a cutoff of 26, correlating with enhanced OS in patients with zero to one pathologically involved node. Cheng et al. [30] set a cutoff of 36.5 based on a significant difference in the total number of LNY between survival and expired groups.

Zhuge et al. [31] identify a cutoff of 19 for correct staging and 15 for improved OS. Kuo et al. [32] establish two distinct cutoffs, 16 for cN0 and 26 for cN + patients.

Additionally, various other studies [13, 14, 33–36] consistently identify 18 as the cutoff, associating it with better survival outcomes.

Only one of these studies identified a cutoff of LNs in the case of bilateral ND. Using a bilateral LNY of ≥ 24 for 5-year OS and ≥ 26 for 5-year DFS gave significantly increased rate advantages of 64 and 56%, respectively (both p < 0.0001) [37].

### Bilateral neck dissection

In four studies, adjustments for bilateral NDs involved calculating the mean number of LNs from both sides [13, 14, 37]. Additionally, Kuo et al. [32] and Divi et al. [14] took a different approach by excluding patients who underwent bilateral NDs from their analyses. Lee et al. [28] specifically focused on the ipsilateral tumor side in instances of bilateral NDs. However, the other studies did not provide information regarding the bilaterality of the performed NDs.

### Type of neck dissection

The predominant consideration in most studies was SND. Notably, Koehler et al. [18] distinguished between radical neck dissection (RND) and selective neck dissection (SND), reporting varying LNY (RND: 6–116, mean 46.0; SND: 6–87, mean 33.6). Feng et al. [19] also differentiated between modified radical neck dissection (MRND) and SND, indicating different LNY (MRND: mean 30.0; SND: mean 21.4). However, in other studies, the specific type of neck dissection performed was not specified.

### cN0 and cN + 

Thirteen studies considered both cN0 and cN + patients without making any distinctions in the results part [3, 14, 18, 19, 22, 28, 29, 31, 38–42]^.^ Among these, nine studies identified cutoffs associated with better survival outcomes ranging from 18 to 35 LNs (Table 1). In four studies, no useful cutoffs were identified.

**Table 1:** Minimum identified LNY in cN0 and cN + patients without any distinction

References	Year	Primary site	Minimum LNY	Population	Included N stages	Survival outcomes	Statistical analysis
Koehler [18]	2010	oc,or	**35**	608	cN0,cN +	OS	U p = 0.048
Ebrahimi [38]	2014	oc	**18**	1567	cN0,cN +	OS,DSS,DFS	U OS p = 0.009 U DSS p = 0.022 U DFS P = 0.043 M OS HR (CI95% 1.1–3.6) 2.0, p = 0.020 M DSS HR (CI95% 1.1–4.5) 2.2, p = 0.043 M DFS HR (CI95% 1.1–2.8) 1.7, p = 0.040
Divi [7]	2016	l,oc,or,hy	**18**	63,978	cN0,cN +	OS	Mortality HR (CI95% 1.13–1.22) 1.18
Kuo [32]	2016	oc	**16 (cN0)** **26 (cN +)**	13,143	cN0,cN +	OS	Mortality for cN0 HR (CI95% 0.764–0.950) 0.825, p = 0.004 Mortality for cN + HR (CI95% 0.692–0.903) 0.791, p = 0.001
Ho [3]	2017	oc	**35**	14,554	cN0,cN +	OS	U HR (CI95% 0.98–0.99) 0.98, p < 0.001
Lee [28]	2018	oc	**19**	78	cN0,cN +	OS,DSS	OS HR (CI95% 1.39– 20.05) 5.29, p = 0.014 DSS HR (CI95% 1.40–32.77) 6.76, p = 0.018
Zenga [29]	2020	or	**26**	2554	cN0,cN +	OS	OS (group with 0 to 1 pathologically involved node HR (CI95% 020–0–78) 0.29
Butt [22]	2022	l,oc,or,hy,ps	**18**	295	cN0,cN +	OS	
Zhuge [31]	2023	oc	19*	2077	cN0,cN +	OS	

*oc* oral cavity, *or* oropharynx, *l* larynx, *hy* hypopharynx, *ps* paranasal sinuses, *DFS* disease-free survival, *DSS* disease-specific survival, *OS* overall survival, *U* univariate analyses, *M* multivariate analyses, *HR* hazard ratio, *CI* confidence interval. In bold the identified minimum LNY cutoff associated with better survival outcomes proposed by each studies

*19 for the accuracy of nodal staging and 15 for the favorable postoperative prognosis

Only one study distinguished the presentation of results between cN0 patients and cN + patients. Specifically, the cutoff associated with better survival outcomes was 16 for cN0 patients and 26 for cN + patients (Table 1). [32]

### cN0

Eleven studies took into consideration only cN0 patients [20, 21, 25–27, 30, 33, 34, 36, 43, 44]. Among these, in ten studies, the identified cutoffs associated with better survival outcomes ranged from 10 to 36.5 LNs. In only one study no useful cutoffs were identified (Table 2).

**Table 2:** Minimum identified LNY in cN0 patients

References	Year	Primary site	Minimum LNY	Population	Included N stages	Survival outcomes	Statistical analysis
Amar [25]	2012	oc	**30**	143	cN0	DFS	U p = 0.02 M p = 0.77
Lemieux [33]	2015	oc	**22**	4341	cN0	OS	22–35 nodes HR 0.854,p 0 = 0.031 36–98 nodes HR 0.827,p 0 = 0.010
Shah [26]	2015	oc	**10**	81	cN0	OS	U p = 0.006
Tsai [27]	2016	oc	**23**	10,442	cN0	OS	U OS p = 0.0007 M all cause mortality HR (CI95% 0.79–0.81) 0.85, p < 0
Safi [43]	2017	oc	**17**	264	cN0	DFS	
Merz [20]	2018	oc	**15**	157	cN0	OS	U p = 0.001
Zenga [34]	2019	oc	**18**	2851	cN0	OS	OS (DOI < 4 mm group) HR (CI95% 0.54–0.85) 0.67 OS (DOI ≥ 4 mm) HR (CI95% 0.34–0.64) 0.47
Cheng [30]	2020	oc	**36.5**	126	cN0	OS	p = 0.019
Leon [21]	2021	oc,or,hy	**15**	647	cN0	OS	HR (CI 95% 1.88–4.45) 2.9, p = 0.0001
Farrokhian [36]	2023	oc	**18**	523	cN0	DSS, DFS	DSS M HR (CI95% 1.04–2.24) 1.53 DFS M HR (CI95% 1.12–1.92) 1.46

*oc* oral cavity, *or* oropharynx, *hy* hypopharynx, *DFS* disease-free survival, *DSS* disease-specific survival, *OS* overall survival, *U* univariate analyses, *M* multivariate analyses, *HR* hazard ratio, *CI* confidence interval. In bold the identified minimum LNY cutoff associated with better survival outcomes proposed by each studies

Three studies do not specify the clinical lymph node status [19], 35, 37. The identified cutoffs associated with better survival outcomes range from 18 to 26 LNs (Table 3).

**Table 3:** Minimum identified LNY in studies with clinical lymph node status not specified

References	Year	Primary site	Minimum LNY	Population	Survival outcomes	Statistical analysis
Feng [19]	2017	oc	**20**	141	DSS	DSS U 58.0% (LNY > 20) vs 21.0% (LNY < 20), p = 0.021
Gomez [35]	2020	or	**18**	4130	OS, DFS	U OS 91.7% for LNY > 18 vs 84.5% for LNY < 18, p = 0.004) M risk of death (LNY > 18) HR (CI95% 0.29–0–70) 0.45, p = 0.001
Boettcher [37]	2020	l	24 and 26*	37	OS,DFS	OS increased rate advantages of 64%, p < 0.001 DFS increased rate advantages of 56%, p < 0.001

*oc* oral cavity, *or* oropharynx, *l* larynx, *DFS* disease-free survival, *DSS* disease-specific survival, *OS* overall survival, *U* univariate analisys, *M* multivariate analysis, *HR* hazard ratio, *CI* confidence interval. In bold the identified minimum LNY cutoff associated with better survival outcomes proposed by each studies

*Bilateral LNY. 24 for augmented OS and 26 for augmented DFS

### Subsites

Four studies grouped various sites of HNSCC [7, 18, 21, 22], while 15 studies specifically addressed the oral cavity. Within the latter set of studies focusing on the oral cavity, a higher LNY was consistently associated with improved OS [3, 19, 20, 25–28, 30–34, 36, 38, 43].

Conversely, only four studies independently examined laryngeal carcinoma; in three of them, an increased LNY did not demonstrate a statistically significant improvement in survival [40, 42], while in the other one, they stated that using a bilateral LNY of ≥ 24 for 5-year OS and ≥ 26 for 5-year DFS gave significantly increased rate advantages of 64 and 56%, respectively (both p < 0.0001) [37].

Furthermore, only three studies examined oropharynx cancer independently. In two of these studies, higher LNY was associated with better prognosis [29, 35], while in the other one, a decreased LNY did not significantly impair the recurrence risk (p = 0.727) [41]. However, this last piece of information can be traced back to the fact that in this study, the number of patients involved is only 82, so the small sample size may be the reason for the lack of statistical significance.

## Discussion

Numerous studies have emphasized the limitations of the current TNM staging system, in the quest for improved prognostic stratification, factors such as LNR and the absolute number of metastatic lymph nodes have shown promise [6]. These factors are intrinsically tied to ND adequacy during surgical procedures. As a result, beyond refining the TNM classification, there is a need to establish criteria for what constitutes an adequate ND, defining a minimum threshold for the number of nodes to be excised. A definitive consensus regarding the optimal minimum number of lymph nodes to be removed during NDs remains elusive in contemporary literature. Nevertheless, compelling evidence suggests a tangible correlation between a higher count of LNY and improved prognosis, even in cases where all excised lymph nodes exhibit negative pathology [33].

This review highlights the absence of a consensus regarding the minimum LNY in HNSCC, ranging from 10 to 36.5. However, the cutoff value of ≥ 18 nodes resulted in a significant improvement of survival outcomes in six studies [7, 22, 34–36, 38]. Most of studies suggest that a higher LNY is associated with improved OS in head and neck cancer patients. However, there are some exceptions to this trend [19, 37, 42], but this could be due to the limited sample size of these studies.

Furthermore, a significant challenge arises from studies treating LNY as a continuous variable, indicating a direct link between increased LNY and reduced survival rates [38]. Nevertheless, utilizing LNY as a continuous variable may offer little practical benefits in clinical settings or decision-making. Hence, determining a specific cutoff value that significantly impacts prognosis remains crucial.

Most of the included studies outline various potential cutoff points, but none delve into the biological mechanisms explaining why an increase in LNY might lead to an improved prognosis. More research is needed to explore the biological reason behind the observed enhancement in OS following higher LNY counts. One plausible explanation might be that an higher LNY results in fewer residual nodes within the patient. Even smaller-sized nodes left behind could potentially harbor tumor deposits, contributing to recurrent metastatic disease. Therefore, establishing a definitive LNY cutoff presents a challenge within the context of this review, mainly due to the considerable heterogeneity evident among the selected studies.

Furthermore, it is crucial to consider the broader array of factors influencing LNY in ND, including patient-specific characteristics, treatment history, the extent of ND, and the methods employed in pathological examination. Another consideration is the potential correlation between LNY and the surgeon's expertise. For instance, the number of lymph nodes removed in colorectal surgery is linked to the surgeon's experience level. Extrapolating this to HNSCC could suggest that more proficient head-neck surgeons might excel in meticulous dissection across all neck levels, potentially impacting LNY counts [45].

In a study conducted by Holcomb et al. [46], it is highlighted that LNY is independently influenced by several factors (such as previous radiotherapy or previous surgical treatment, body mass index, the number of dissected lymph node levels, and the presence of pathologically positive lymph nodes) including the pathological technique used to analyze the specimen. This study introduced a novel pathology protocol, consisting of including residual adipose tissue into tissue blocks for analysis, independently contributing to increased LNY.

While surgical quality has traditionally been deemed the primary determinant of LNY, this study shows numerous external factors beyond the surgeon's control that can significantly affect LNY outcomes, making identifying a cutoff even more challenging [46].

Moreover, most of the included studies demonstrating the correlation between LNY and survival outcomes and support for its inclusion as a measure of surgical quality rely on database data without insights into the specifics of pathological assessment methodologies. Therefore, ensuring accurate and consistent pathological assessment of ND specimens becomes imperative.

The primary limitation of this review stems from the substantial heterogeneity among the included studies. Despite their exclusive focus on HNSCC, variability exists in the specific subsites involved, each requiring the dissection of different lymph node levels based on distinct indications. This variability directly influences the number of dissected levels and the total count of removed lymph nodes. Data regarding the lymph nodal level involved in each neck dissection and the number of lymph node retrieved for each level lacked in the majority of the articles reviewed; further investigation assessing the minimum number of lymph node that should be dissected at each level are needed and might be explored in future studies.

Additionally, differences in the approach to cN0 versus cN + cases further contribute to fluctuations in LNY. For instance, ECS or liquefied lymph nodes in cN + cases could reduce the count of removed lymph nodes [47].

To enhance data comparability, standardization of the ND technique and the subsequent pathological processing of the specimen becomes imperative. Achieving such standardization is essential to ensure consistency and reliability in assessing LNY across different studies and clinical contexts.

This review underscores the association between increased LNY and enhanced prognosis in patients diagnosed with HNSCC. The LNY not only serves as a prognostic indicator, but also mirrors the efficacy of neck treatment, directly impacting staging decisions and recommendations for potential additional adjuvant therapies after surgery.

Nevertheless, a greater depth of evidence is imperative to fortify these observations. This can be achieved through prospective studies designed to minimize inherent biases and comprehensively consider all the options. The gathering of data from well-structured, expansive studies holds promise for reinforcing and refining our understanding of the role of LNY. This, in turn, could significantly contribute to optimizing therapeutic approaches and ensuring more accurate prognostic evaluations for patients affected by HNSCC. Therefore, further studies are required to determine whether there is an optimal LNY cut-off for prognostication and suggest which patients may benefit from adjunctive therapy and optimize follow-up protocols.

## Declarations

### Data availability

The data used in this study are available upon request from the corresponding author.
